# Engineering High-Amylose and High-Dietary-Fibre Barley Grains Through Multiplex Genome Editing of Four Starch-Synthetic Genes

**DOI:** 10.3390/foods14132319

**Published:** 2025-06-30

**Authors:** Qiang Yang, Jean-Philippe Ral, Qiantao Jiang, Zhongyi Li

**Affiliations:** 1 Institute of Quality Standard and Testing Technology Research, Sichuan Academy of Agricultural Sciences, Chengdu 610066, China; qiangyi551724@126.com; 2 State Key Laboratory of Crop Gene Exploration and Utilisation in Southwest China, Triticeae Research Institute, Sichuan Agricultural University, Chengdu Campus, Chengdu 611130, China; 3 Agriculture and Food, Commonwealth Scientific and Industrial Research Organisation, Black Mountain, Canberra, ACT 2601, Australia

**Keywords:** *Hordeum vulgare*, CRISPR/Cas9, multiplex genome editing, resistant starch, starch properties

## Abstract

Barley, rich in beneficial ingredients, has been recognised as a healthy food and is widely used in the production of healthy foods for humans. The current study identified a new barley mutant with the *SSIIa*, *SSIIIa*, *SBEIIa*, and *SBEIIb* genes inactivated in the genome-edited offspring of targeted mutagenesis of starch synthetic genes using multiplex genome editing. The grain compositions and starch properties of the *ssIIa/ssIIIa/sbeIIa/sbeIIb* mutant were analysed and compared with the corresponding parameters of *ssIIa*, *ssIIIa*, *sbeIIa/sbeIIb*, *ssIIa/sbeIIa/sbeIIb*, and non-genome-edited lines (NE), respectively. *ssIIa/ssIIIa/sbeIIa/sbeIIb* exhibited the highest contents of β-glucan and amylose content among all mutants and NE, but not the most prominent in resistant starch, fructan, and fibre contents. The loss of *SSIIa*, *SSIIIa*, *SBEIIa*, and *SBEIIb* genes also resulted in significant changes in starch properties. This study enriched the genotypes of healthy barley and provided a theoretical basis for improving barley quality.

## 1. Introduction

Barley (*Hordeum vulgare* L.) is a type of conventional, cultivated cereal, accounting for approximately 12% of the world’s total cereal production [1,2]. Historically, barley grains were primarily used as feed grains and in the brewing and distilling industry, such as beer brewing and other food production [3]. More recently, attention has turned to the nutritional and chemical properties of the biologically active constituents in barley grain. Barley is considered the most suitable grain for the human diet due to the high dietary fibre content and the high proportion of soluble viscous fibre components. The hypoglycaemic and hypocholesterolemic functions of β-glucan also suggest that barley-based foods are good for human health [4].

The primary reserve storage polysaccharide of barley grain is starch, which accounts for about 60% of the dry weight of barley grain. Typically, the total starch in barley grain contains about 25% amylose, with the remaining portion being amylopectin [5,6]. However, unique barley materials were created in previous studies; high-amylose barley contained more than 80% amylose content, and waxy barley had about 100% amylopectin content [2,7]. The biosynthesis of starch in barley grains was like that in other cereals, which is coregulated by enzymes named ADP-glucose Pyrophosphorylase (AGPase), granule-bound starch synthase (GBSS), soluble starch synthase (SSS), starch branching enzyme (SBE), and starch debranching enzyme (DBE). Each was divided into several types based on their roles in starch biosynthesis [8]. GBSS was essential for the biosynthesis of amylose, and SSS and SBE enzymes were coordinated to regulate amylopectin synthesis. Previous studies have indicated that the amylose (Am) and amylopectin (Ap) contents, as well as the Am/Ap ratio, can influence the quality of starch-based products. Recently, considerable attention has been focused on a specific type of starch known as resistant starch (RS), which remains undigestible both in vitro and in vivo. Products based on RS have been identified as a healthy food source for individuals with diabetes. Previous studies have indicated that cereals with higher amylose content typically have a higher resistant starch (RS) content than regular barley [9]. Therefore, barley with high amylose (RS) content makes a positive contribution to the production of healthy food.

Genetic improvement is the most effective means of breeding plants with target phenotypes in crop breeding for various crops. Mutants with superior agronomic traits were obtained by large-scale screening, and the beneficial phenotypes were transferred to other cultivated varieties for crop improvement by crossing. Chemical mutagenesis is a common and effective method for inducing mutants with superior agronomic traits. It has been successfully used for breeding barley with high amylose content, wheat with high resistance to stem rust, and rice with high nutritional value [10,11,12]. However, the mutations induced by chemical mutagenesis were uncertain, and identifying mutants with targeted genotypes and phenotypes is laborious and time-consuming. A succession of backcrosses was required to reduce the other mutation sites in the mutant backgrounds induced by chemical mutagenesis before they could be used for crop improvement. These deficiencies limited the large-scale use of chemical mutagenesis in crop genetic improvement. Genetic engineering technology is an effective means to realise the improvement of donor cereals. The targets and timelines of genetic engineering means are more predictable than other standard genetic improvement methods. To date, it has been successfully utilised to enhance the yield, quality, and resistance of crops [13]. Although the public worries about the bio-safety of GM products, the convenience and effectiveness of transgenic technology vigorously promote its application in crop improvement.

The CRISPR/Cas9 system is a valuable genetic tool for inducing targeted mutagenesis. A genome-editing construct containing the Cas9 gene and guide RNA expression cassettes was first transformed into the donor plant. The targeted DNA double-strand breaks (DSBs) were induced in the transformed plant cells, and indels were introduced during the non-homologous end joining (NHEJ) or homologous recombination (HR) repair processes. Homozygous mutants of target genes can be easily identified in the offspring of mutant plants using molecular biological methods, and transgene-free mutants can be obtained through segregation in self-crossing or backcrossing populations of genome-edited lines [14]. CRISPR/Cas9 gene-editing technology made it easy to realise targeted mutagenesis, and the identification process was more effective and efficient due to the lack of random mutagenesis, saving time for laborious screening. So far, CRISPR/Cas9 is recognised as the most effective technology for studying the function of endogenous genetic factors in cereals [15,16,17]. Additionally, the CRISPR/Cas9 genome-editing technology is a powerful tool for producing mutations in multiple genes within a single transgenic event. It avoids the complicated process of multiple genetic transformation work or the time-consuming process of crosses among multiple mutants to create polygenic mutants. Due to its simple operations, multiplex gene editing technology has become the most frequently used method for creating targeted mutants. To date, it has been successfully applied to the improvement of rice, wheat, barley, and other crops [13]. Our earlier published paper demonstrated that 14 out of 155 T_0_ barley plants harboured heterozygous or homozygous mutations for all four genes, including three plants with homozygous mutations for one gene and two plants with homozygous mutations for two genes [18]. All four genes were fully inactivated in the quadruple mutant, as they all lost their protein expression, as shown below.

In a previous study, a barley mutant with the *ssIIa* gene inactivated was created using chemical mutagenesis and targeted genome editing. The starch properties and the mechanism of the increased resistant starch (RS) content in SSIIa null mutants were analysed. In addition, the *ssIIa* null mutant, BARLEYmax, with high resistant starch content, has been widely used worldwide. Experiments on pigs and rats have shown that feeding BARLEYmax benefits the animals’ health [19,20,21], suggesting that BARLEYmax food containing high RS content can improve human health. Additionally, the multiplex genome-editing system was utilised for the targeted editing of genes related to amylopectin synthesis in barley. Various mutants, genotyped as ssIIa, ssIIIa, ssIVa, sbeIIa, sbeIIb, ssIIa/ssIIIa, ssIIa/ssIVa, sbeIIa/sbeIIb, ssIIa/ssIIIa/ssIVa, and ssIIa/sbeIIa/sbeIIb, were obtained through a single transgene event via Agrobacterium-mediated genetic transformation of barley immature embryos. Grain ingredients and starch properties of different mutants were analysed to study the effect of *SSIIa*, *SSIIIa*, *SSIVa*, *SBEIIa*, and *SBEIIb* in barley starch biosynthesis [18]. The studies above systematically analysed the functions of amylopectin synthesis-related genes in producing RS in barley grains. However, creating barley mutants with different genotypes remains the most effective way to further investigate the function of related genes and their synergistic relationship. In the current study, a four-gene mutant, genotyped as *ssIIa/ssIIIa/sbeIIa/sbeIIb*, was identified in the progeny of genome-edited mutants created in a previous study. The grain morphology, various composition parameters, and starch properties of *ssIIa/ssIIIa/sbeIIa/sbeIIb* were analysed and compared to the corresponding parameters in *ssIIa*, *ssIIIa*, *sbeIIa/sbeIIb*, and *ssIIa/sbeIIa/sbeIIb*, respectively. These results further demonstrated the effect of *ssIIa*, *ssIIIa*, *sbeIIa*, and *sbeIIb* in the biosynthesis of barley starch.

## 2. Materials and Methods

### 2.1. Plant Materials

The genome-edited barley T_0_ lines, induced by *Agrobacterium tumefaciens*-mediated transformation of recombinant Agrobacterium with a genome-editing construct, were used to identify mutants with target genotypes. The procedures were described in detail previously for constructing the multiplex genome-editing construct, barley transformation, screening of transgenic positive T_0_ plants, and genotyping of T_0_ plants [18]. All T_0_ plants were grown in a glasshouse at the plant growth facility of CSIRO Agriculture and Food (Canberra, ACT, Australia) at 25 °C under natural light conditions. The transgenic donor Golden Promise and non-genome-edited lines grown side by side with mutant lines were used as controls.

### 2.2. Identification of ssIIa/ssIIIa/sbeIIa/sbeIIb Mutants

The genome-edited transgenic T_0_ lines containing mutations in the *SSIIa*, *SSIIIa*, *SBEIIa*, and *SBEIIb* genes were selected to identify *ssIIa/ssIIIa/sbeIIa/sbIIb* mutants. Based on the number of mutant target genes contained in the genome-edited T_0_ plant, different numbers of dried T_1_ grains were randomly selected from the progenies of each selected T_0_ mutant. All of them were germinated and transplanted into the soil, and the genomic DNA of each T_1_ plant was extracted. The genotype of each T_1_ plant was analysed using KASP markers, which were designed based on the genotype of the corresponding T_0_ plant at each sgRNA site. Based on the results of KASP detection, the T_1_ generation plants with target genotypes were selected for subsequent studies. The genotypes were then confirmed by Sanger sequencing with the primer pairs to identify the corresponding sgRNA site [18]. The T_1_ plants with genotype *ssIIa/ssIIIa/sbeIIa/sbeIIb* were transplanted into large pots, and mature grains were harvested for the subsequent study.

### 2.3. Grain Weight

The mature grains of T_2_ plants with genotype *ssIIa/ssIIIa/sbeIIa/sbeIIb* were harvested and dried in a 37 °C oven. The 50-grain weight of *ssIIa/ssIIIa/sbeIIa/sbeIIb* was measured and converted to a 1000-grain weight. The average grain weight was calculated as the mean of triplicate measurements of 1000 grains for each T_2_ mutant plant.

### 2.4. Microscopic Examination of Barley Grains

Fifty grains of each *ssIIa/ssIIIa/sbeIIa/sbeIIb* mutant plant were randomly selected to measure the average grain length, width, and area. Grains were randomly spread on the sample plate, leaving sufficient space between seeds. The original images of grain morphological parameters were recorded using the Epson Perfection V330 Photo scanner (Epson, Suwa, Nagano, Japan). The data on grain length, width, and area of each grain were analysed by the supporting software CSIRO GrainScan version 3. Additionally, a camera and a Leica microscope digital camera (M80, Leica, Wetzlar, Germany) were used to record the grain shape in multiple grains and individual grains, respectively. The mature grains of non-genome-edited lines (NE) were used as the negative control.

### 2.5. Starch Extraction

The mature grains of *ssIIa/ssIIIa/sbeIIa/sbeIIb* mutant and NE lines were used to extract the starch granules. The procedure has been described in detail [18]. Dried mature grains were milled by ESPE CapMixTM (model 3M, Seefeld, Germany), and treated with Hydrochloric acid (0.02 M), NaOH solution (0.2M), 0.1M Tris-HCl buffer (pH 7.6, containing 0.5% sodium metabisulphite), protease K (LS004224, Worthington Biochemical, Lakewood, NJ, USA), and Percol (17-0891-01, GE Healthcare, Mascot, NSW, Australia) in the successive procedure. The starch suspension was used to analyse the starch granule particle size directly, and the remaining part was dried in a freeze-dryer (BenchTop Pro with Omnitronics, SP SCIENTIFIC, Warminster, PA, USA) and stored at 4 °C for analysis of the starch composition and properties.

### 2.6. SDS-PAGE and Immunoblotting Analysis

The purified and dried starch granules were used to extract granule-bound proteins (GBPs) SSIIa, SBEIIa, and SBEIIb. A 4 mg sample of starch was accurately weighed and homogenised with 60 µL of extraction buffer containing 5% SDS, 10% glycerol, 5% β-mercaptoethanol, 50 mM Tris buffer (pH 6.8), and bromophenol blue in a 1.5 mL centrifuge tube. The starch-containing tubes were vortexed, boiled for 10 min, and then centrifuged at 13,000 rpm (model 5424, Eppendorf AG, Hamburg, Germany) for 20 min at 4 °C. For SDS-PAGE analysis, a 25 µL supernatant was pipetted onto a mini PAGE gel (NuPAGETM 4–12% Bis-Tris Gel, NP0322BOX, Thermo Fisher, Santa Clara, CA, USA). For analysing the protein expression of SSIIIa, one or two immature endosperms were homogenated with 200 µL extraction buffer containing 50 mM KPi pH 7.5, 5 mM EDTA, 20% glycerol, 1 mM PIC, and 10 mM DTT in a 1.5 mL centrifuge tube using a pestle, and then were centrifuged at 14,000 rpm for 15 min at 4 °C immediately. The supernatant was transferred to a new Eppendorf tube, and 5 µL of it were used for quantifying the protein content using a Coomassie Plus Protein Assay Reagent kit (23238, Thermo Fisher, Santa Clara, CA, USA). The protein samples loaded onto the same gel were diluted to the same concentration with the extraction buffer. An equal volume of 2 × SDS loading buffer was added to each tube and mixed well using a pipette containing 100 mM Tris-HCl, 20% glycerol, 4% SDS, and 0.2% bromophenol blue. The protein-containing tubes were vortexed, boiled for 5 min, and transferred to ice immediately. In total, 25 µL of the mixture were pipetted onto a mini PAGE gel for SDS-PAGE analysis.

The electrophoresis and immunoblotting procedures were the same for analysing both GBPs and soluble starch synthases. Electrophoresis was performed in MOPS–Tris–SDS buffer (50 mM MOPS, 50 mM Tris, 0.1% SDS, and 1 mM EDTA) using a Mini gel tank (Life Technologies, Thermo Fisher, Santa Clara, CA, USA) with a constant voltage of 100 V for 140 min.

After electrophoresis, the gels were removed gently, rinsed in water, soaked in ethanol (20%) for ten minutes, and rinsed in Milli Q water. A preheated iBlot 2 Dry Blotting System (IB1001, Invitrogen, Life Technologies, Thermo Fisher, Santa Clara, CA, USA) was used to transfer proteins from the gel onto a PVDF membrane using the P_0_ program. The PVDF membranes were soaked in TTBS buffer (20 mM Tris, pH 7.4, 150 mM NaCl, 2.5 mM KCl, and 0.1% Tween 20) containing 3% skim milk immediately, with slow shaking at 30 rpm at room temperature for 1 h. The PVDF membranes were removed with tweezers and soaked in a TTBS buffer containing 0.2% skim milk and a suitable concentration of the tested primary antibody against the corresponding target protein. The membranes were incubated overnight at 4 °C with slow shaking at 30 rpm. The membrane was washed with TTBS for 30 min, during which the TTBS was changed twice. The membrane was incubated in TTBS containing 0.2% skim milk and a suitable concentration of goat anti-rabbit IgG (H + L) with HRP conjugate (170-6515, BIO-RAD, USA) at room temperature for 30 min with slow shaking at 30 times per minute. The washing step was repeated, and the membranes were washed three times with TTBS for 10 min each time. The membranes were lifted with tweezers, wrapped with Super Signal West Pico Plus solution (34580, Thermo Fisher, USA), and imaged using myECL imager (62236x, Thermo Fisher, Santa Clara, CA, USA) [6].

### 2.7. Microscopic Imaging and Particle Size Analysis of Barley Starch Granules

A scanning electron microscope (SEM) (EVO LS15, ZEISS, Jena, Germany) was used to observe and record the morphology of the barley starch granules. The purified and dried barley starch granules of different mutants and controls were fixed on an aluminium stub and coated with gold particles. Images were taken using the SEM at a voltage of 20 kV with a 1000× magnification.

The purified starch slurries were used to analyse the starch granule size distribution using Mastersizer 3000 (Malvern Instruments, Malvern, UK). The procedures were performed according to the manufacturer’s instructions. The starch granules were divided into A-type (>10 μm) and B-type (1–10 μm) according to the cut-off diameters, and the volume percentages of A and B types were calculated, respectively. The A-type starch granules were also subdivided into three groups: A1 granule (10–20 μm), A2 granule (20–60 μm), and A3 granule (60–120 μm). The differences were calculated in the B-type, A1, A2, and A3 granule compositions between *ssIIa/ssIIIa/sbeIIa/sbeIIb* and other mutants [6].

### 2.8. Measurement of Grain Ingredients

A series of Megazyme’s commercial kits (No. K-TSTA; No. K-RSTAR; No. K-BGLU; No. K-FRUCHK; No. K-TDFR, Megazyme, Wicklow, Ireland) were used to analyse the contents of total starch, resistant starch, β-glucan, fructan, and total dietary fibre in *ssIIa/ssIIIa/sbeIIa/sbeIIb* mutant grains, respectively. The dried mature barley grains were milled to pass a 0.5 mm screen using ESPE CapMixTM (model 3M, Seefeld, Germany). The barley wholemeal was dried in a 37 °C oven until there was no further weight loss, and the dried wholemeal was weighed according to the requirements of different Megazyme kits for grain content analyses. The procedures followed the manufacturer’s instructions, and each parameter of a different sample was performed in triplicate.

The apparent amylose content of *ssIIa/ssIIIa/sbeIIa/sbeIIb* mutant barley was assayed using the iodine-binding method [22], described in detail in our previous study [23].

### 2.9. Analyses of Starch Structure and Properties

The amylopectin chain length distribution of debranched *ssIIa/ssIIIa/sbeIIa/sbeIIb* mutant starch was analysed using a fluorescence-activated capillary electrophoresis (7100 Capillary Electrophoresis, Agilent Technologies, Santa Clara, CA, USA). The procedures were performed with minor modifications to the previous method [24]. About ten milligrams of purified starch for each test sample were weighed and dissolved in 375 μL distilled water and 25 μL NaOH (2M) in a 1.5 mL centrifuge tube. The starch samples were boiled for 5 min and then cooled at room temperature. Then, solutions were added to the sample tubes in turn, including 16 μL of glacial acetic acid, 50 μL of sodium acetate (1M), 500 μL distilled water, and 5 μL of the iso-amylase enzyme (Megazyme, Wicklow, Ireland), and the tubes were thoroughly vortexed after each solution was added. The tubes were incubated in a 37 °C water bath for 2 h and then boiled for 10 min. A 100 μL mixture was transferred into a new 1.5 mL Eppendorf tube and dried in a speed vacuum (SAVANT DNA 120 SpeedVac Concentrator, Thermo Scientific, Scoresby, VIC, Australia). A 7 μL APTS (8-amino-1,3,6,-pyrenetrisulfonic acid) solution (containing 5 mg APTS labelling dye in 48 μL 15% acetic acid) and 7 μL sodium cyanoborohydride solution (6.3 mg dissolved in 100 µL distilled water) were added to dissolve the dried sample, and the sample tubes were incubated for ten to 14 h in a 50 °C bio-shaker with 350 rpm shaking speed. After incubation, 60 μL of distilled water were added to each tube to dilute the sample. The tubes were then boiled for 1.5 min and immediately spun for 30 s in a microcentrifuge. The mixture was filtered through a Wizard miniColumn (Promega part A7211) and used for fluorescence-activated capillary electrophoresis. The results were analysed by the supporting software of the P/ACE 5510 capillary electrophoresis system (Beckman, Brea, CA, USA) with agron-LIF detection.

Based on the structural differences between amylose and amylopectin, the amylose content of the *ssIIa/ssIIIa/sbeIIa/sbeIIb* mutant was measured using high-performance liquid chromatography (HPLC). The procedures were performed according to the method described by Batey and Curtin with a minor change [25]. For each sample, approximately 5 milligrams of purified starch were weighed and dissolved in 500 μL UDMSO (90% DMSO containing 0.6 M Urea) in a 1.5 mL tube. The mixture was then incubated overnight at 80 °C in a bio-shaker at 400 rpm. After 500 μL of 80 °C preheated distilled water were added to dilute the sample, 100 μL of the mixture were loaded for HPLC to analyse the amylose content of the branched starch sample. The remaining mixture was cooled to 40 °C in a 40 °C bio-shaker. In total, 100 μL of acetate buffer (0.5 M, pH 4.0) and 8 μL of normal iso-amylase enzyme (Megazyme, Wicklow, Ireland) were added in turn, and the mixture was incubated at 40 °C with 800 rpm shaking for 3.5 h. The samples were then heated up to 80 °C and incubated for one hour. After 0.2 g of resin were added, the tubes were incubated at 60 °C, shaken at 1000 rpm for 30 min, and centrifuged at 5000 rpm for 5 min. The mixture was filtered through a 0.45 μm filter membrane and used to analyse the amylose content of the debranched starch sample.

The swelling power and solubility of *ssIIa/ssIIIa/sbeIIa/sbeIIb* mutant barley starch were measured with a 20 mg swelling test method. The moisture content of barley starch was equilibrated by leaving the dried sample with the lid open at room temperature for 10 to 16 h. The labelled 2 mL screw-cap tubes were dried in a 75 °C oven for one hour with the lids open. After closing the lids, the tubes were transferred to a desiccator and cooled down to room temperature. The tube weights were weighed and recorded as W1. About 20 mg of starch were weighed and added to the corresponding tube, and the exact weight of starch was recorded as W2. One millilitre of distilled water was added to dissolve the starch, and the tubes were incubated in a 92.5 °C water bath for 30 min. Twenty iterations of inverted mixings were performed in the first minute, followed by two iterations of inverted mixings at 1.5 min, 2 min, 3 min, 4 min, 5 min, 7.5 min, 10 min, 15 min, and 25 min. Tubes were transferred into a 20 °C water bath and cooled for 3 min, followed by two rounds of inverted mixing at both 0 and 1.5 min time points. The tubes were immediately centrifuged for 10 min at 14,000 rpm, and the supernatant was removed using a pipette. The tubes with open lids were placed in a 75 °C oven to dry the samples for one hour. After closing the lids, the tubes were transferred to a desiccator and cooled to room temperature. The tube weights were then recorded as W3. After opening the lids, the sample tubes were placed in a 75 °C oven and dried to a constant weight in a 37 °C oven. The tubes were transferred into a desiccator with lids closed for cooling down for more than half an hour, then weighed once more and recorded as W4. The swelling power and solubility of barley starch were calculated according to the published formulas, respectively. The formulas were: swelling power = (W3 −W1) / (W2 − 8% × W2) and solubility = (W1 + W2 − W4) / (W2 − 8% × W2/100) × 100.

The Differential Scanning Calorimeter (DSC 8000, PerkinElmer, Buckinghamshire, UK) was used to analyse the calorimetry profiles of *ssIIa/ssIIIa/sbeIIa/sbeIIb* mutant starch. Approximately 60 mg of starch were weighed after the moisture content equilibration, and the exact weight was recorded as W1. The starch was then mixed with X μL of distilled water (the volume of water was calculated using the formula X = 2 × (W1 − W1 × 8%)). Three repeats of the same premixed sample (approximately 50 mg) were sealed in DSC pans, and the weights of the premixed samples were accurately recorded. The DSC pans containing the premixed samples were equilibrated at room temperature for seven days. The pans were used for DSC measurements directly, and the program was performed following the parameters with a 10 °C increase per minute and varying in radians from 20 °C to 140 °C. An empty DSC pan was treated with the same parameters as a reference. The DSC pans were stored at room temperature for one month and used to measure the amylose-lipid dissociation enthalpies with the same parameters. At last, the DSC thermogram was analysed using the instrument software.

### 2.10. Statistical Analyses

Triplicate measurements were taken for grain size, grain weight, the particle size of starch granules, calorimetry profiles, starch swelling power and solubility, and the contents of total starch, apparent amylose, resistant starch, β-Glucan, fructan, and the total dietary fibre. The data were analysed and expressed as the mean ± standard deviation. The data for NE, *ssIIa*, *ssIIIa*, *sbeIIa/sbeIIb*, and *ssIIa/sbeIIa/sbeIIb* were published in our previous study [18]. The differences between the tested samples with different genotypes were analysed using IBM SPSS Statistics 21 and Minitab 19.

## 3. Results

### 3.1. Genotypes of ssIIa/ssIIIa/sbeIIa/sbeIIb Mutants

**Figure 1:**
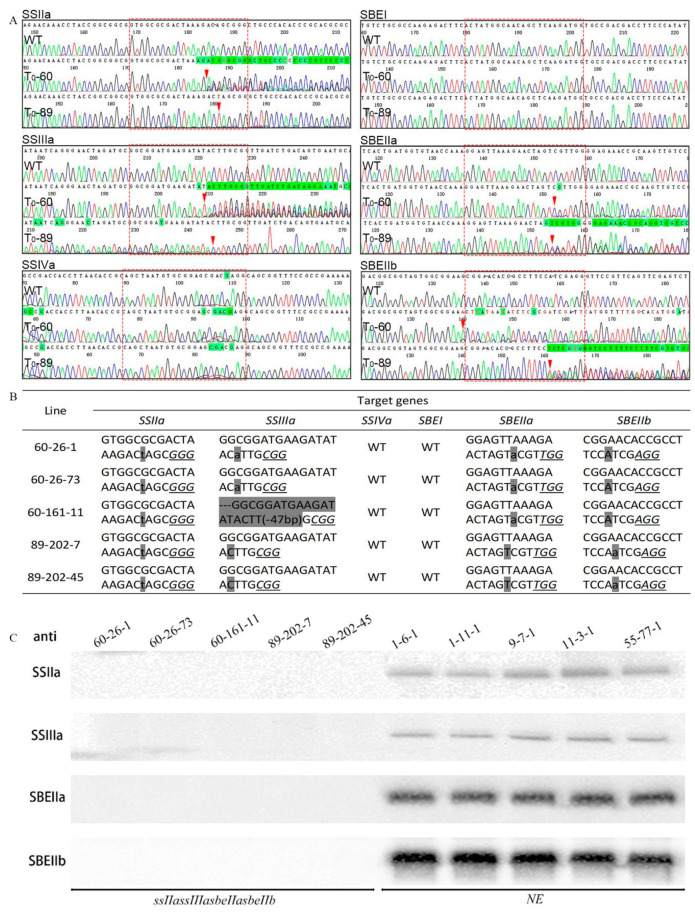
Genotype and immunoblot analyses of *ssIIa/ssIIIa/sbeIIa/sbeIIb* mutant plants. (**A**) The sequence results of T_0_-60 and T_0_-89 mutant lines. WT, Golden Promise. The box, indicated by red dashed lines in each image, represents the gRNA region of the corresponding gene. The red arrows indicate the mutation site in the T_0_ plants at the corresponding gRNA site. (**B**) The mutation genotypes of the target genes in the selected homozygous T_2_ mutants. The line numbers of T_2_ plants are marked on the left side, and the names of the target genes are marked at the top of the sequences. GP represents the non-transgenic barley ‘Golden Promise’; WT indicates that no mutation was detected in the transgenic plant at the corresponding gRNA site. Italic letters with underlines indicate the PAM sequences of gRNA sites. The lowercase letters on a grey background represent the insertions, and the capital letters on a grey background represent the deletions of the nucleotides. (**C**) Starch granules from five independent T_3_ mutant lines and NE were used to extract the GBPs. The protein bands detected by various antibodies are indicated on the left side of each image. NE, non-genome-edited line.

In our previous study, 155 T_0_ barley plants were obtained by transforming barley immature embryos with a recombinant Agrobacterium carrying a genome-editing construct. Of these, 152 were alive, and 113 were identified as genome-edited mutants containing one to six targeted gene mutations [18]. Based on the sequencing results of these T_0_ mutant plants, both T_0_-60 and T_0_-89 were identified as genome-edited mutants that contained heterozygous mutations for the *SSIIa*, *SSIIIa*, *SBEIIa*, and *SBEIIb* genes (Figure 1A). Approximately 400 of the T_1_ offspring of T_0_-60 and T_0_-89 were germinated and planted for the screening of *ssIIa/ssIIIa/sbeIIa/sbeIIb* homozygous mutants. KASP genotyping was used to analyse the genotype of each T1 plant preliminarily, and the genotypes of the ssIIa/ssIIIa/sbeIIa/sbeIIb mutants identified by KASP analysis were subsequently verified using Sanger sequencing. Finally, two T_1_ progenies of the T_0_-60 line, T_1_ 60-26 and T_1_60-161, and one T_1_ offspring of the T_0_-89 line, T_1_ 89-202, were identified as *ssIIa/ssIIIa/sbeIIa/sbeIIb* heterozygous mutants.

The genotypes of T_2_ generation plants were analysed to illustrate the inheritance of mutations in different generations. Five T_2_ generation plants (T_2_ 60-26-1 and T_2_ 60-26-73, T_2_ 60-161-11, T_2_ 89-202-7, and T_2_ 89-202-45) were randomly selected from the progeny of T_1_ 60-26, T_1_ 60-161, and T_1_ 89-202, respectively. The genotypes were analysed by Sanger sequencing and are shown in Figure 1B. T_2_ 60-26-1 and T_2_ 60-26-73 mutants had a T insertion in the *SSIIa* gene, an A insertion in the *SSIIIa* gene, an A insertion in the *SBEIIa* gene, and an A deletion in the *SBEIIb* gene. The genotype of T_2_ 60-161-11 was identical to that of T_2_ 60-26-1 and T_2_ 60-26-73, except for a 47 bp deletion in the SSIIIa gene. Both T_2_ 89-202-7 and T_2_ 89-202-45 contained a T insertion in the *SSIIa* gene, a C deletion in the *SSIIIa* gene, a T deletion in *SBEIIa*, and an A insertion in the *SBEIIb* gene. It was found that all the T_2_ plants had the same genotype as the corresponding T_1_ generation.

### 3.2. The Protein Expression Levels of the Mutation Genes in the ssIIa/ssIIIa/sbeIIa/sbeIIb Mutants

The protein expression levels of *SSIIa*, *SSIIIa*, *SBEIIa*, and *SBEIIb* genes in the *ssIIa/ssIIIa/sbeIIa/sbeIIb* mutants were analysed by immunoblotting using antibodies specific to each gene. The purified starch granules of mature barley grains were used to extract GBPs to analyse the expression level of *SSIIa*, *SBEIIa*, and *SBEIIb*. Immature endosperms at 15 days post-anthesis were used to extract proteins for analysing the expression levels of the soluble starch synthetase gene *SSIIIa*. Equal amounts of protein were loaded into different holes on one gel, and Western blotting was used to display the results and quantify the expression level of each mutation gene. The immunoblot assays using specific antisera revealed no detectable protein expression encoded by the *SSIIa*, *SSIIIa*, *SBEIIa*, and *SBEIIb* genes in *ssIIa/ssIIIa/sbeIIa/sbeIIb* mutants (Figure 1C).

### 3.3. The Morphology and Sizes of the ssIIa/ssIIIa/sbeIIa/sbeIIb Mutant Grains

The camera images and stereo microstructure of *ssIIa/ssIIIa/sbeIIa/sbeIIb* T_3_ mature grains were captured using a digital camera and a digital microscope camera system, respectively. The images of non-genome-edited barley grains resembled those of the mature grains of the transgenic donor, Golden Promise, as reported in previous studies. All the *ssIIa/ssIIIa/sbeIIa/sbeIIb* mutant grains showed a shrunken endosperm, with an unfilled central region of the dorsal side of barley grains, and the invagination of the abdominal side was more severe than that of the non-genome-edited mature grains (Supplementary Figure S1).

The average 1000-grain weight of *ssIIa/ssIIIa/sbeIIa/sbeIIb* mutant grain was 30.80 ± 0.20 g, which was significantly lower than that in the *NE*, *ssIIIa*, *sbeIIa/sbeIIb*, and *ssIIa/sbeIIa/sbeIIb*, respectively, and there were no significant differences between *ssIIa/ssIIIa/sbeIIa/sbeIIb* and *ssIIa* mutants in the 1000-grain weight (Supplementary Figure S2A).

The dimensions of *ssIIa/ssIIIa/sbeIIa/sbeIIb* mutant grains were measured using the scanning equipment, and the size parameters were analysed. The average grain length of *ssIIa/ssIIIa/sbeIIa/sbeIIb* mutant grains was 8.60 ± 0.17 mm, which was significantly shorter than that in NE, ssIIIa, sbeIIa/sbeIIb, and ssIIa/sbeIIa/sbeIIb. The average grain width of *ssIIa/ssIIIa/sbeIIa/sbeIIb* (3.18 ± 0.05 mm) was significantly lower than that in NE, *ssIIa*, and *ssIIIa*. There were no significant differences in the grain width among *ssIIa/ssIIIa/sbeIIa/sbeIIb*, *sbeIIa/sbeIIb*, and *ssIIa/sbeIIa/sbeIIb* mutants. For the grain area, there was no significant difference between *ssIIa/ssIIIa/sbeIIa/sbeIIb* (21.60 ± 0.54 mm^2^) and *ssIIa/sbeIIa/sbeIIb*. In addition, the grain area of *ssIIa/ssIIIa/sbeIIa/sbeIIb* was significantly smaller than that in NE and mutants with other genotypes (Supplementary Figure S2B).

### 3.4. The Composition of ssIIa/ssIIIa/sbeIIa/sbeIIb Mutant Grain

**Figure 2:**
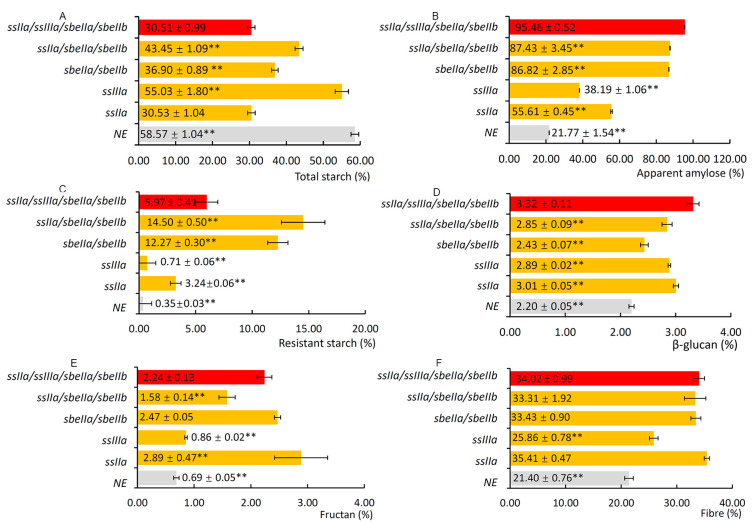
Compositions of mutant barley grains. (**A**) Total starch content. (**B**) Apparent amylose content. (**C**) Resistant starch content. (**D**) β-glucan content. (**E**) Fructan content. (**F**) Dietary fibre content. The numbers in columns represent the means of five biological replicates and the standard errors of the corresponding parameters, respectively. The grey columns represent the contents of the different components of the non-genome-edited barley grains. The red columns represent the contents of the *ssIIa/ssIIIa/sbeIIa/sbeIIb* mutant grains. The yellow columns represent the content of corresponding components in the grains of *ssIIa*, *ssIIIa*, *sbeIIa/sbeIIb*, and *ssIIa/sbeIIa/sbeIIb* mutants. The data in grey font in the columns indicate that the data were published in a previous study [18]. Asterisks indicate the significant differences between the *ssIIa/ssIIIa/sbeIIa/sbeIIb* mutant grains and other mutant grains, and non-genome-edited grains determined by Student’s *t*-test (** at *p* < 0.01).

The contents of total starch, apparent amylose, resistant starch, β-glucan, fructan, and total dietary fibre in *ssIIa/ssIIIa/sbeIIa/sbeIIb* mutant grains were measured and compared with those in NE, *ssIIa*, *ssIIIa*, *sbeIIa/sbeIIb*, and *ssIIa/sbeIIa/sbeIIb*. The total starch content of the *ssIIa/ssIIIa/sbeIIa/sbeIIb* mutant was 30.51 ± 0.99%, about half of that in NE, which was significantly lower than that in *ssIIIa*, *sbeIIa/sbeIIb*, and *ssIIa/sbeIIa/sbeIIb*. However, there was no significant difference between *ssIIa/ssIIIa/sbeIIa/sbeIIb* and *ssIIa* mutants in total starch content (Figure 2A). The apparent amylose content in each mutant was much higher than that in NE. The apparent amylose content of *ssIIa/ssIIIa/sbeIIa/sbeIIb* mutant (95.46 ± 0.52%) was significantly higher than that in *ssIIa*, *ssIIIa*, *sbeIIa/sbeIIb*, and *ssIIa/sbeIIa/sbeIIb*, which was four times higher than that in NE (Figure 2B). The resistant starch content of *ssIIa/ssIIIa/sbeIIa/sbeIIb* mutant was 5.97 ± 0.41%, which was about twenty times that of NE, two times that of the *ssIIa* mutant, and eight times that of the *ssIIIa* mutant, respectively, and about one-half and two-fifths that of *sbeIIa/sbeIIb* and *ssIIa/sbeIIa/sbeIIb*, respectively. In general, the content of resistant starch in *ssIIa/ssIIIa/sbeIIa/sbeIIb* was significantly higher than that in NE, *ssIIa*, and *ssIIIa*, and significantly lower than that in *sbeIIa/sbeIIb* and *ssIIa/sbeIIa/sbeIIb.* In addition, the data showed that the resistant starch content in mutants with SBEIIa and SBEIIb genes knocked out was significantly higher than that in other mutants (Figure 2C). The contents of β-glucan, fructan, and fibre in *ssIIa/ssIIIa/sbeIIa/sbeIIb* mutants were 3.32 ± 0.11%, 2.24 ± 0.13%, and 34.02 ± 0.99%, respectively, which were significantly higher than the contents of corresponding components in NE. The β-glucan content of the *ssIIa/ssIIIa/sbeIIa/sbeIIb* mutant was considerably higher than that in *ssIIa*, *ssIIIa*, *sbeIIa/sbeIIb*, and *ssIIa/sbeIIa/sbeIIb* (Figure 2D). The fructan content in *ssIIa/ssIIIa/sbeIIa/sbeIIb* was significantly higher than that in *ssIIIa* and *ssIIa/sbeIIa/sbeIIb*, significantly lower than that in *ssIIa*. There was no significant difference between *sbeIIa/sbeIIb* and *ssIIa/ssIIIa/sbeIIa/sbeIIb* in the fructan content (Figure 2E). The *ssIIa/ssIIIa/sbeIIa/sbeIIb* mutant had a similar level of fibre content as other mutants, except that in *ssIIIa*, which was significantly higher than that in NE and *ssIIIa* mutants (Figure 2F).

### 3.5. The Amylose Content of ssIIa/ssIIIa/sbeIIa/sbeIIb Mutant Grains

**Figure 3:**
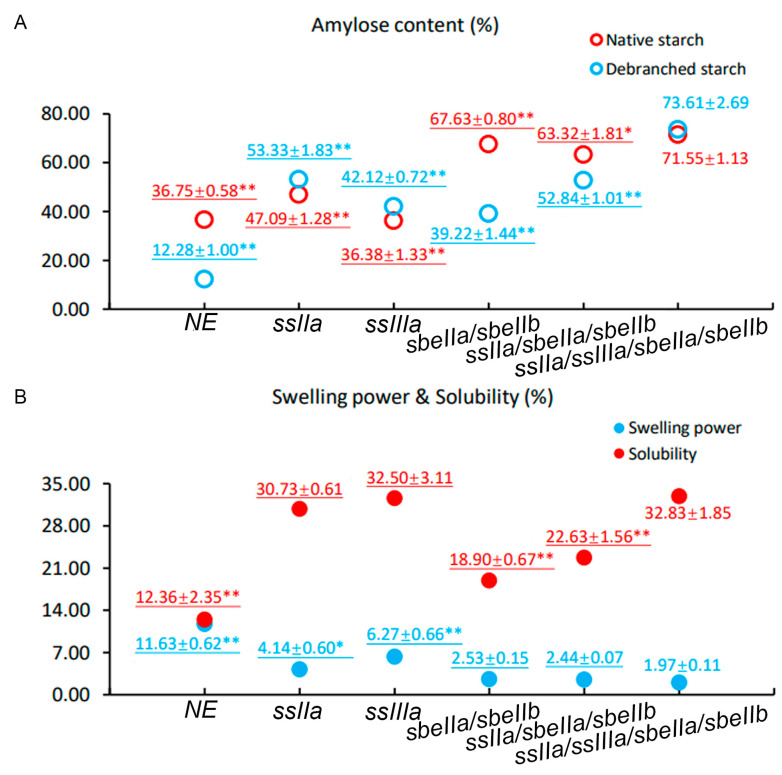
Amylose content, starch swelling power, and solubility of T_3_ mutants and NE barley grains. (**A**) Amylose content of starch from T_3_ mutants and NE grains measured by HPLC. The data in red and blue fonts indicate the amylose content of native and debranched starch from barley grains of the same genotype, respectively. (**B**) Swelling power and solubility of starch from T_3_ mutants and NE grains. The data in red and blue fonts indicate the swelling power and solubility of the same starch sample, respectively. The genotypes of barley lines are labelled on the x-axis. The numbers above the circles (hollow or solid) represent the means of five biological replicates, along with their standard errors. The data underlined indicate that the data were published in a previous study [18]. NE, non-genome-edited lines. Asterisks indicate the significant difference between the *ssIIa/ssIIIa/sbeIIa/sbeIIb* mutant grains and other mutant grains, and NE determined by Student’s *t*-test (** at *p* < 0.01).

The amylose content (Am) of *ssIIa/ssIIIa/sbeIIa/sbeIIb* was measured based on the microstructure difference between amylose and amylopectin. The amylose content of *ssIIa/ssIIIa/sbeIIa/sbeIIb* in native and debranched starch was analysed using HPLC, respectively. In the native starch, the Am in *ssIIa/ssIIIa/sbeIIa/sbeIIb* (71.55 ± 1.13%) was approximately twice that in NE and *ssIIIa*, and significantly higher than that in *ssIIa*, *sbeIIa/sbeIIb*, and *ssIIa/sbeIIa/sbeIIb*. The Am of debranched *ssIIa/ssIIIa/sbeIIa/sbeIIb* starch (73.61 ± 2.69%) was significantly higher than that in *ssIIa*, *ssIIIa*, *sbeIIa/sbeIIb*, and *ssIIa/sbeIIa/sbeIIb*, and six times higher than that in debranched NE starch. For *ssIIa/ssIIIa/sbeIIa/sbeIIb* mutant, the Am of debranched starch was higher than that in native starch; a similar situation was found in the Am of *ssIIa* and *ssIIIa* mutants. However, the Am of debranched starch was lower than that in native starch in *sbeIIa/sbeIIb*, *ssIIa/sbeIIa/sbeIIb*, and NE. The difference in Am in native and debranched starch of *ssIIa/ssIIIa/sbeIIa/sbeIIb* was less than that of NE and all the other mutants (Figure 3A).

### 3.6. Starch Swelling Power and Solubility

The swelling power and solubility of the purified *ssIIa/ssIIIa/sbeIIa/sbeIIb* starch were measured. The result showed that the swelling power of the *ssIIa/ssIIIa/sbeIIa/sbeIIb* mutant was 1.97 ± 0.11%, significantly lower than that in NE, *ssIIa*, and *ssIIIa* null mutants. There were no significant differences among *sbeIIa/sbeIIb*, *ssIIa/sbeIIa/sbeIIb*, and *ssIIa/ssIIIa/sbeIIa/sbeIIb* in starch swelling power with the inactivated *SBEIIa* and *SBEIIb* genes. The solubility of *ssIIa/ssIIIa/sbeIIa/sbeIIb* mutant was 32.83 ± 1.85%, about three times that of NE, which was higher than that of NE, *sbeIIa/sbeIIb*, and *ssIIa/sbeIIa/sbeIIb* significantly. However, there were no significant differences among *ssIIa*, *ssIIIa*, and *ssIIa/ssIIIa/sbeIIa/sbeIIb* mutants in the starch solubility (Figure 3B).

### 3.7. Chain Length Distribution of Debranched Starch

**Figure 4:**
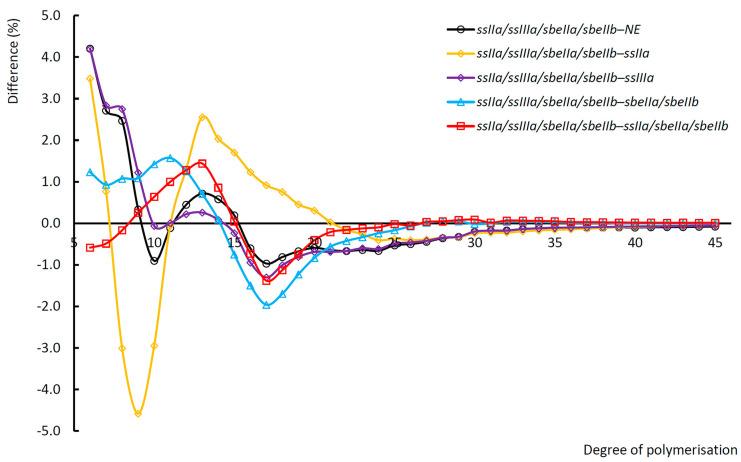
Differential patterns between *ssIIa/ssIIIa/sbeIIa/sbeIIb* and other mutants, and NE in the chain length distribution of debranched starches. The black, yellow, purple, blue, and red lines represent the differences between *ssIIa/ssIIIa/sbeIIa/sbeIIb* mutant and NE, *ssIIa*, *ssIIIa*, *sbeIIa/sbeIIb*, and *ssIIa/sbeIIa/sbeIIb* mutant barley, respectively. NE, non-genome-edited line. The x-axis represents the DP sizes.

The chain length distribution of *ssIIa/ssIIIa/sbeIIa/sbeIIb* debranched starch was analysed by fluorescence-activated capillary electrophoresis (FACE). The differences in chain length distributions between *ssIIa/ssIIIa/sbeIIa/sbeIIb* null mutant and other mutants were calculated. The data of NE, *ssIIa*, *ssIIIa*, *sbeIIa/sbeIIb*, and *ssIIa/sbeIIa/sbeIIb* were subtracted from the *ssIIa/ssIIIa/sbeIIa/sbeIIb*, respectively, as shown in Figure 4. Based on the published division criteria, the amylopectin molecule chains were divided into four groups named short, medium, long, and very long chains, with chain lengths of DP6-12, DP13-24, DP25-36, and DP > 36, respectively. The differences between the *ssIIa/ssIIIa/sbeIIa/sbeIIb* null mutant and other mutants in each group were digitised and presented in Table 1. The results showed that *ssIIa/ssIIIa/sbeIIa/sbeIIb* had a higher proportion of short chains than that in NE and *ssIIIa*, and lower proportions of the medium, long, and very long chains than that in NE and *ssIIIa* null mutants. The *ssIIa/ssIIIa/sbeIIa/sbeIIb* had higher proportions of medium chains and lower proportions of short, long, and very long chains than those in the *ssIIa* null mutant. The proportions of short and medium chains in *ssIIa/ssIIIa/sbeIIa/sbeIIb* were higher and lower than those in both *sbeIIa/sbeIIb* and *ssIIa/sbeIIa/sbeIIb* mutants, respectively. There were no noticeable differences among *sbeIIa/sbeIIb*, *ssIIa/sbeIIa/sbeIIb*, and *ssIIa/ssIIIa/sbeIIa/sbeIIb* for the proportions of long and very long chains.

### 3.8. The Granule Morphology and Particle Size Distribution of Barley Starch

**Figure 5:**
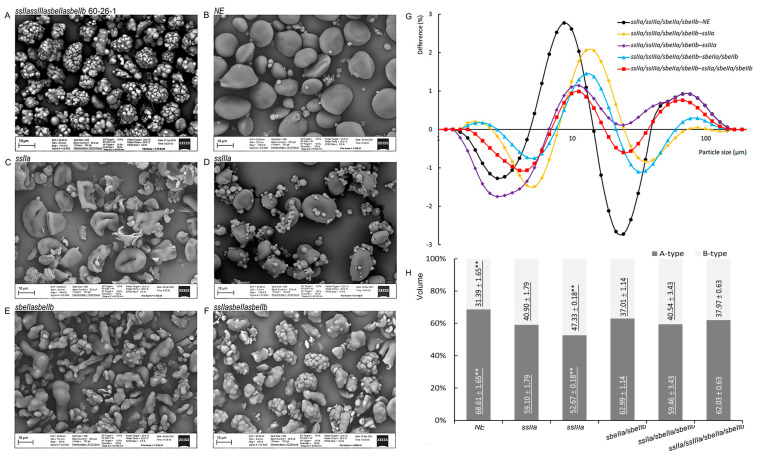
Starch granule morphologies and compositions in mutant barley grains. Starch granule morphologies are shown in *ssIIa/ssIIIa/sbeIIa/sbeIIb* null mutant (**A**); non-genome-edited (NE) control (**B**); *ssIIa* null mutant (**C**); *ssIIIa* null mutant (**D**); *sbeIIa/sbeIIb* null mutant (**E**); *ssIIa/sbeIIa/sbeIIb* null mutant (**F**). (**G**) shows differential patterns between *ssIIa/ssIIIa/sbeIIa/sbeIIb* and other mutants, and NE in the starch granule size distribution. The black, yellow, purple, blue, and red lines represent the differences between *ssIIa/ssIIIa/sbeIIa/sbeIIb* mutant and NE, *ssIIa*, *ssIIIa*, *sbeIIa/sbeIIb*, and *ssIIa/sbeIIa/sbeIIb* mutant barley lines, respectively. NE, non-genome-edited lines. (**H**) shows starch granule compositions of starches from T_3_ mutants and NE grains. The columns in dark grey and light grey represent the percentage volumes of A-type and B-type starch granules, respectively. The mean ± SD data in the columns were calculated from five replicates. The data underlined indicates that the data were published in a previous study. Asterisks indicate a significant difference between the *ssIIa/ssIIIa/sbeIIa/sbeIIb* mutant and other mutants and NE determined by Student’s *t*-test (* at *p* < 0.05; ** at *p* < 0.01). NE, non-genome-edited lines.

The purified starches extracted from NE, *ssIIa*, *ssIIIa*, *sbeIIa/sbeIIb*, *ssIIa/sbeIIa/sbeIIb*, and *ssIIa/ssIIIa/sbeIIa/sbeIIb* were used to analyse the morphology of starch granules (Figure 5A-F). The results showed that the morphologies of NE, *ssIIa*, *ssIIIa*, *sbeIIa/sbeIIb*, and *ssIIa/sbeIIa/sbeIIb* starch granules were consistent with the published starch microstructure. The morphologies of NE starch granules were typical of barley starches, containing a certain amount of A-type and B-type starch granules with spherical granular morphology. There were apparent differences between mutants and NE in the shapes and compositions of starch granules. The A-type starch granules in *ssIIa* showed an apparent hollow surface compared to those of normal starch in NE. The *ssIIIa* had smaller A-type starch granules and more B-type granules than those in NE. Many distorted A-type starch granules with stick-like, smooth surfaces were observed in *sbeIIa/sbeIIb*, and some of them exhibited concave surfaces. *ssIIa/sbeIIa/sbeIIb* had new kinds of spherical A-type starch granules consisting of several small smooth surface granules with or without clear boundaries, and the sizes of those altered granules were like the typical A-type starch granules in NE. All the changes in the size and composition of starch granules in *ssIIa*, *ssIIIa*, *sbeIIa/sbeIIb*, and *ssIIa/sbeIIa/sbeIIb* were consistent with the starch microstructure published in our previous study (Figure 5B–F). Compared with NE and other mutants, *ssIIa/ssIIIa/sbeIIa/sbeIIb* had altered the microstructure of starch granules. *ssIIa/ssIIIa/sbeIIa/sbeIIb* did not have any B-type starch granules, and all A-type starch granules had abnormal morphologies. A small number of granules were altered with a microstructure similar to those observed in *sbeIIa/sbeIIb*, and the remaining granules looked like those in *ssIIa/sbeIIa/sbeIIb*, except that those granules consisted of smaller granules with more explicit boundaries (Figure 5A; Supplementary Figure S3).

The particle size distributions of starch granules of *ssIIa/ssIIIa/sbeIIa/sbeIIb* were analysed using Mastersizer 3000. The differences in the particle size distributions of starch granules between *ssIIa/ssIIIa/sbeIIa/sbeIIb* and other mutants were calculated. The percentage of volume of granules with different particle sizes in NE, *ssIIa*, *ssIIIa*, *sbeIIa/sbeIIb*, and *ssIIa/sbeIIa/sbeIIb* was subtracted from the data of *ssIIa/ssIIIa/sbeIIa/sbeIIb*, respectively, as shown in Figure 5G. The starch granules in NE and barley mutants were divided into A- and B-type granules based on their diameter. The volume percentage of A- and B-type starch granules in NE and mutants with different genotypes was calculated, respectively. The results showed that the volume percentage of A- and B-type starch granules in *ssIIa/ssIIIa/sbeIIa/sbeIIb* was 62.03 ± 0.63% and 37.97 ± 0.63%, respectively (Figure 5H). The volume percentage of A-type starch granules in *ssIIa/ssIIIa/sbeIIa/sbeIIb* was significantly higher than that in *ssIIIa* but significantly lower than that in NE. The percentage of the B-type granules in *ssIIa/ssIIIa/sbeIIa/sbeIIb* was significantly higher than that in NE, whereas it was significantly lower than that in *ssIIIa*. There were no significant differences among *ssIIa/ssIIIa/sbeIIa/sbeIIb*, *ssIIa*, *sbeIIa/sbeIIb*, and *ssIIa/sbeIIa/sbeIIb* in the volume percentage of A- and B-type starch granules.

Figure 5G also showed that inactivating the target genes in different mutants had a dramatic effect on the composition of starch granules (Table 2). There are two peaks above (approximately 10–20 μm and 60–120 μm) and one below (20–60 μm) the zero line of the X-axis, named A1, A2 and A3. The proportion of B-type starch granules in *ssIIa/ssIIIa/sbeIIa/sbeIIb* was higher than that in NE, but lower than that in other mutants. For the A-type granules, the *ssIIa/ssIIIa/sbeIIa/sbeIIb* mutant increased A1 size granules compared to NE and all other mutants. However, the *ssIIa/ssIIIa/sbeIIa/sbeIIb* mutant reduced the proportions of A2 size granules compared with NE. The proportion of A2 and A3 granules in *ssIIa/ssIIIa/sbeIIa/sbeIIb* was reduced compared with *ssIIa* and *sbeIIa/sbeIIb*. Whereas the proportion of A2 and A3 granules in *ssIIa/ssIIIa/sbeIIa/sbeIIb* was increased compared with *ssIIIa* and *ssIIa/sbeIIa/sbeIIb*, respectively.

### 3.9. Thermal Properties

The thermal properties of the starch from *ssIIa/ssIIIa/sbeIIa/sbeIIb* mutant were analysed using DSC, and the gelatinisation temperature of *ssIIa/ssIIIa/sbeIIa/sbeIIb* was calculated and shown in Table S1. For the gelatinisation peak, the onset, peak, and final temperatures of *ssIIa/ssIIIa/sbeIIa/sbeIIb* were significantly higher than those in NE and ssIIa, but significantly lower than those in *ssIIa/sbeIIa/sbeIIb*, respectively. The onset temperature of *ssIIa/ssIIIa/sbeIIa/sbeIIb* was substantially higher than that in *ssIIIa*, and there were no significant differences between *ssIIa/ssIIIa/sbeIIa/sbeIIb* and *ssIIIa* in the peak and end temperatures. Otherwise, the onset and peak temperature of *ssIIa/ssIIIa/sbeIIa/sbeIIb* were significantly lower than that in *sbeIIa/sbeIIb*, but there was no significant difference between *ssIIa/ssIIIa/sbeIIa/sbeIIb* and *sbeIIa/sbeIIb* in the end temperature. The enthalpy of *ssIIa/ssIIIa/sbeIIa/sbeIIb* was significantly lower than that in NE and *ssIIIa*. There were no significant differences between *ssIIa/ssIIIa/sbeIIa/sbeIIb* and *ssIIa*, *sbeIIa/sbeIIb*, and *ssIIa/sbeIIa/sbeIIb*. For the gelatinisation temperature of the amylose-lipid dissociation peak, there were no significant differences between *ssIIa/ssIIIa/sbeIIa/sbeIIb* and *sbeIIa/sbeIIb* in onset, peak, and end temperatures. The data for *ssIIa/ssIIIa/sbeIIa/sbeIIb*, *ssIIa*, and *ssIIa/sbeIIa/sbeIIb* showed no significant differences in the onset and peak temperatures. For other parameters, the data for *ssIIa/ssIIIa/sbeIIa/sbeIIb* were all significantly lower than those in other samples with different genotypes. There were no significant differences among *ssIIa/ssIIIa/sbeIIa/sbeIb* mutant and NE, as well as other mutants for enthalpy.

## 4. Discussion

### 4.1. Cas9-Mediated Mutagenesis Produced Barley Mutants

The efficient CRISPR/Cas9 genome editing system for barley has been successfully established, and barley genetic engineering mutants have been produced using CRISPR/Cas9 genome editing technology to target and induce mutations at the promoter region of the *HvPAPhy_a* gene and the coding region of the *HvPM19* gene, respectively [26,27]. The CRISPR/Cas9 system facilitated the study of endogenous genetic factors in barley by enabling targeted mutagenesis of specific genes. The barley mutants with the lost ADPG binding structure of starch synthase IIa (SSIIa) were induced through targeted mutagenesis of SSIIa by CRISPR/Cas9 genome editing [23]. The novel starch phenotype in the *ssIIa* null mutant barley significantly increased amylose and resistant starch contents, and the starch structure and functions were changed considerably compared to the control, which was consistent with the early reported barley *ssIIa* null mutant, Himaraya292 (BARLEYmax), induced by the chemical mutagenesis [11]. In addition, the Cas9-mediated multi-gene editing system was successfully utilised for barley gene editing of seven starch biosynthetic genes, with six being edited efficiently in a single transgenic event. A range of homozygous monogenic, double, and triple null mutants were identified in the progenies of mutant lines, and the novel phenotypes of different mutants were analysed [18]. This study identified a homozygous four-gene mutant *ssIIa/ssIIIa/sbeIIa/sbeIIb* from the same population of genome-edited mutants. Theoretically, a larger-scale screening can identify more mutants with different genotypes, containing one to six mutated genes, from the same population. Both the Cas9-mediated single- and multi-gene editing systems are excellent tools for creating mutants to study the functional elucidation of causal genetic variants and elements in barley. However, the acquisition of homozygous mutants during the multi-gene editing procedure depends on the self-segregation of T_0_ mutants. The isolation of one genome-edited target site was consistent with Mendelian genetics for the heterozygous mutation of one target gene [28]. Selecting mutations that target multiple genes in a single multi-gene editing event would significantly increase the complexity of identifying homozygous mutant progenies. It is necessary to balance the experimental requirements with the feasibility of identifying homozygous mutant progenies.

### 4.2. Mutated SSIIa, SSIIIa, SBEIIa, and SBEIIb Genes in Barley Led to the Novel Starch Phenotype and Grain Compositions

The starch properties and grain compositions of the *ssIIa/ssIIIa/sbeIIa/sbeIIb* mutant were analysed and compared with NE and other mutants. The morphology and composition of the starch granules in *ssIIa/ssIIIa/sbeIIa/sbeIIb* changed more than those in the *ssIIa*, *ssIIIa*, *sbeIIa/sbeIIb*, and *ssIIa/sbeIIa/sbeIIb*. No A-type starch granules with normal-sharp in *ssIIa/ssIIIa/sbeIIa/sbeIIb* were observed as those in NE, and no stick-like starch granules were present in ssIIa/ssIIIa/sbeIIa/sbeIIb as those in *sbeIIa/sbeIIb* mutant. The starch granules in *ssIIa/ssIIIa/sbeIIa/sbeIIb* were polyhedron spherical granules with separated small granules or a convex surface, and the polymerised small granules were more obvious than those in *ssIIa/sbeIIa/sbeIIb* [18]. The percentage volume of the A1, A2, A3, and B-type starch granules in *ssIIa/ssIIIa/sbeIIa/sbeIIb* was changed compared with that in NE and *ssIIa*, *ssIIIa*, *sbeIIa/sbeIIb*, and *ssIIa/sbeIIa/sbeIIb*, showing the changes of starch granule morphology and compositions in *ssIIa/ssIIIa/sbeIIa/sbeIIb. ssIIa/ssIIIa/sbeIIa/sbeIIb* mutant reduced B granules and increased A1 size granules compared with the other four mutants, reflecting the effect of different target genes in the biosynthesis of starch granules.

A previous study indicated that starch with a higher percentage of DP13-24 average amylopectin branch chain length exhibits higher thermal properties [29]. This study analysed the chain-length distribution and thermal properties of *ssIIa/ssIIIa/sbeIIa/sbeIIb* using FACE and DSC, respectively. The *ssIIa/ssIIIa/sbeIIa/sbeIIb* line had a lower portion of DP13-24 chains than that in NE, which was associated with significantly higher gelatinisation temperatures than those in NE. This result contradicted the previous conclusion but was similar to that in *ssIIa/sbeIIa/sbeIIb* mutants, which contained a lower percentage of DP13-24 and higher gelatinisation temperatures than NE [18].

The mutant grain compositions showed that the *ssIIa/ssIIIa/sbeIIa/sbeIIb* had significantly higher contents of apparent amylose, resistant starch, β-glucan, fructan, and fibre than those in NE. The contents of apparent amylose and β-glucan in *ssIIa/ssIIIa/sbeIIa/sbeIIb* were even substantially higher than those in *ssIIa*, *ssIIIa*, *sbeIIa/sbeIIb*, and *ssIIa/sbeIIa/sbeIIb*, respectively. However, *ssIIa/ssIIIa/sbeIIa/sbeIIb* contained significantly lower resistant starch content than those in *sbeIIa/sbeIIb* and *ssIIa/sbeIIa/sbeIIb*. It did not consistently support the published conclusion that resistant starch content was positively correlated with the amylose content [9]. Similar results were found in the analysis of the grain composition of *ssIIa/ssIIIa/ssIVa*, which contained a significantly higher amylose content; however, the resistant starch content was considerably lower than that in *ssIIa* [18]. One of the potential reasons was that the inactivation of SSIIIa leads to a reduction in the proportion of chains of DP > 37 in starch [6], which may reduce the proportion of the long chains and affect its resistant starch content starch in the quadruple mutant compared to those in *sbeIIa/sbeIIb* and *ssIIa/sbeIIa/sbeIIb*. Such a reduction of the long chains was not detected in the chain length distribution of debranched starches between *ssIIa/ssIIIa/sbeIIa/sbeIIb* and *ssIIa/sbeIIa/sbeIIb* mutants in our current study. Further studies are needed to understand the mechanisms by which the *ssIIIa* mutation affects RS content in the quadruple mutant. In our earlier published paper, the amylose content assay methods were compared for barley starches from different gene mutants, including apparent amylose in native starch and debranched starch, and the iodine-binding method [30]. In general, high-amylose starches from all mutant barley lines yielded high amylose values using all three methods. However, different mutants had high amylose phenotypes through various mechanisms. The *amo1/ssIIa* double mutant produced long-chain amylose molecules, and the *sbeIIa/sbeIIb* double mutant reduced short chains of amylopectin. Although the iodine-binding method can estimate the high amylose content phenotype, combining apparent amylose in native starch and debranched starch helps explain whether high amylose starch is produced from an increased proportion of long-chain amylose or a reduced proportion of amylopectin. The grain compositions suggested that *ssIIa/ssIIIa/sbeIIa/sbeIIb* is one of the most promising candidate genotypes for breeding healthy barley.

### 4.3. Cas9-Mediated Multi-Gene Editing System Helps to Improve the Health Quality of Barley

Like other cereal grains, starch is the principal storage polysaccharide of barley grains, comprising about 25% amylose and 75% amylopectin. Previous studies have shown that the quality of cereal-based foods is significantly influenced by the properties of starch [6,11,31]. The high-amylose starch, with a high amylose/amylopectin ratio, also contained a high resistant starch content, which is beneficial for health [32]. The amylose and resistant starch contents of the *ssIIa* null barley mutant were significantly higher than those in the control, which proved useful to animal health [1,4,20,32,33]. The barley genetically engineered mutants with single, double, or triple null mutations of the starch biosynthetic genes were induced using the CRISPR/Cas9 multiplex genome-editing system [18]. The *sbeIIa/sbeIIb* and *ssIIa/sbeIIa/sbeIIb* mutants contained around four and 35 times more apparent amylose and resistant starch contents than NE, respectively. These mutants would benefit human health and are even superior to the *ssIIa* null barley mutant. However, the high amylose/resistant starch contents negatively affect grain-eating and cooking qualities [15,34,35]. In the current study, the *ssIIa/ssIIIa/sbeIIa/sbeIIb* barley mutant was identified from the offspring of a genome-edited mutant induced by a multiplex genome-editing system. The apparent amylose and resistant starch content of *ssIIa/ssIIIa/sbeIIa/sbeIIb* was significantly higher than that in the control. Moreover, the apparent amylose content of *ssIIa/ssIIIa/sbeIIa/sbeIIb* was significantly higher than that in *ssIIa*, *ssIIIa*, *sbeIIa/sbeIIb*, and *ssIIa/sbeIIa/sbeIIb*. However, the resistant starch content of *ssIIa/ssIIIa/sbeIIa/sbeIIb* was significantly higher than that in *ssIIa* and *ssIIIa*, but considerably lower than that in *sbeIIa/sbeIIb* and *ssIIa/sbeIIa/sbeIIb*. Our study enriched the understanding of the various types of amylose and resistant starch contents in barley. Moreover, the contents of β-glucan, fructan, and fibre of *ssIIa/ssIIIa/sbeIIa/sbeIIb* were significantly increased compared with those in the control, showing potential outstanding health effects. The present study identified a new type of barley with potential health benefits, which provides theoretical guidance and genetic resources for barley breeding. Although no in vivo studies have been done in the quadruple mutant, CSIRO’s early in vivo work on the *ssIIa* mutant barley grain showed the health benefits of whole grains, fructan, and β-glucan of the mutant barley [20,21,32,36,37,38]. Future in vivo studies may be conducted to validate the health benefits of the quadruple mutant, as demonstrated in the *ssIIa* mutant barley grain, if the quadruple mutant is selected for breeding and commercialisation.

## 5. Conclusions

In conclusion, this study generated the barley *ssIIa/ssIIIa/sbeIIa/sbeIIb* null mutant from the genome-edited offspring of targeted mutagenesis barley lines. The grain composition and starch properties were analysed systematically for *ssIIa/ssIIIa/sbeIIa/sbeIIb* null barley grains. In comparison with the related single, double, and triple-gene barley mutants, *ssIIa/ssIIIa/sbeIIa/sbeIIb* showed prominent advantages in the contents of amylose and β-glucan and some advantages in the contents of resistant starch, fructan, and fibre compared to some of the related mutants. This study indicated that *ssIIa/ssIIIa/sbeIIa/sbeIIb* null mutant barley is a new germplasm for cultivating healthy barley. However, several future works need to be conducted. Firstly, transcriptomic or proteomic research may be conducted to further understand the mechanisms by which gene mutations in the quadruple mutant affect different grain traits, such as high amylose, β-glucan, and fructan contents. Secondly, the in vivo work may also be conducted to validate the health benefits of the quadruple mutant, if required for breeding and commercialisation. Lastly, further research will be conducted on the gene editing of barley grain genes to seek optimised genetic combinations for target grain traits that improve health benefits.

## Figures and Tables

**Table 1:** Differences in chain length distribution between *ssIIa/ssIIIa/sbeIIa/sbeIIb* and other barley mutants.

Genotype	Differences in Chain Length Distribution (%)			
	DP6-12	DP13-24	DP25-36	DP > 36
*ssIIa/ssIIIa/sbeIIa/sbeIIb* − *NE*	9.11	−4.77	−3.19	−1.15
*ssIIa/ssIIIa/sbeIIa/sbeIIb* − *ssIIa*	−5.04	9.16	−3.20	−0.92
*ssIIa/ssIIIa/sbeIIa/sbeIIb* − *ssIIIa*	11.14	−7.21	−3.05	−0.88
*ssIIa/ssIIIa/sbeIIa/sbeIIb* − *sbeIIa/sbeIIb*	8.66	−8.71	0.01	0.04
*ssIIa/ssIIIa/sbeIIa/sbeIIb* − *ssIIa/sbeIIa/sbeIIb*	2.02	−2.64	0.46	0.16

**Table 2:** Differential compositions of starch granules between *ssIIa/ssIIIa/sbeIIa/sbeIIb* and other barley mutants.

Genotype	Differential Compositions of Starch Granules (%)			
	B Granule (1–10 μm)	A_1_ Granule (10–20 μm)	A_2_ Granule (20–60 μm)	A_3_ Granule (60–120 μm)
*ssIIa/ssIIIa/sbeIIa/sbeIIb* − *NE*	1.06	3.45	−7.92	3.41
*ssIIa/ssIIIa/sbeIIa/sbeIIb* − *ssIIa*	−8.95	12.03	−1.79	−1.27
*ssIIa/ssIIIa/sbeIIa/sbeIIb* − *ssIIIa*	−15.51	6.98	5.09	3.41
*ssIIa/ssIIIa/sbeIIa/sbeIIb* − *sbeIIa/sbeIIb*	−4.18	9.91	−5.49	−0.25
*ssIIa/ssIIIa/sbeIIa/sbeIIb* − *ssIIa/sbeIIa/sbeIIb*	−9.16	5.7	1.92	1.53

## Data Availability

The original contributions presented in the study are included in the article/Supplementary Materials, further inquiries can be directed to the corresponding authors.

## Supplementary Materials

The following supporting information can be downloaded at https://www.mdpi.com/article/10.3390/foods14132319/s1. Figure S1. Stereoscopic and camera photographs of *ssIIa/ssIIIa/sbeIIa/sbeIIb* T_3_ mutant grains. Figure S2. Grain weight and dimensions of T3 mutant barley grains. Figure S3. Starch granule morphologies in different *ssIIa/ssIIIa/sbeIIa/sbeIIb* mutant lines. Table S1. Differences in starch gelatinisation temperatures between ssIIa/ssIIIa/sbeIIa/sbeIIb and other barley mutants.
