# Supplementary material for: Engineering High-Amylose and High-Dietary-Fibre Barley Grains Through Multiplex Genome Editing of Four Starch-Synthetic Genes
Source: Foods. 2025 Jun 30;14(13):2319. doi: 10.3390/foods14132319 (PMC12249315; doi:10.3390/foods14132319)
Supplement: Supplementary file 1 [file foods-14-02319-s001.zip › foods-3652555-supplementary.pdf]

# Engineering High-Amylose and High-Dietary-Fibre Barley Grains Through Multiplex Genome Editing of Four Starch-Synthetic Genes

**Qiang Yang [1,2,3], Jean-Philippe Ral [3], Qiantao Jiang [2,*] and Zhongyi Li [3,*]**

[1] Institute of Quality Standard and Testing Technology Research, Sichuan Academy of Agricultural Sciences, Chengdu 610066, China; qiangyi551724@126.com
[2] State Key Laboratory of Crop Gene Exploration and Utilisation in Southwest China, Triticeae Research Institute, Sichuan Agricultural University, Chengdu campus, Chengdu 611130, China
[3] Agriculture and Food, Commonwealth Scientific and Industrial Research Organisation, Black Mountain, Canberra, ACT 2601, Australia; jean.ral@csiro.au
* Correspondence: qiantaojiang@sicau.edu.cn (Q.J.); zonhrc@gmail.com (Z.L.)

## Abstract

Barley, rich in beneficial ingredients, has been recognised as a healthy food and is widely used in the production of healthy foods for humans. The current study identified a new barley mutant with the *SSIIa*, *SSIIIa*, *SBEIIa*, and *SBEIIb* genes inactivated in the genome-edited offspring of targeted mutagenesis of starch synthetic genes using multiplex genome editing. The grain compositions and starch properties of the *ssIIa/ssIIIa/sbeIIa/sbeIIb* mutant were analysed and compared with the corresponding parameters of *ssIIa*, *ssIIIa*, *sbeIIa/sbeIIb*, *ssIIa/sbeIIa/sbeIIb*, and non-genome-edited lines (NE), respectively. *ssIIa/ssIIIa/sbeIIa/sbeIIb* exhibited the highest contents of β-glucan and amylose content among all mutants and NE, but not the most prominent in resistant starch, fructan, and fibre contents. The loss of *SSIIa*, *SSIIIa*, *SBEIIa*, and *SBEIIb* genes also resulted in significant changes in starch properties. This study enriched the genotypes of healthy barley and provided a theoretical basis for improving barley quality.

**Keywords:** *Hordeum vulgare*; CRISPR/Cas9; multiplex genome editing; resistant starch; starch properties

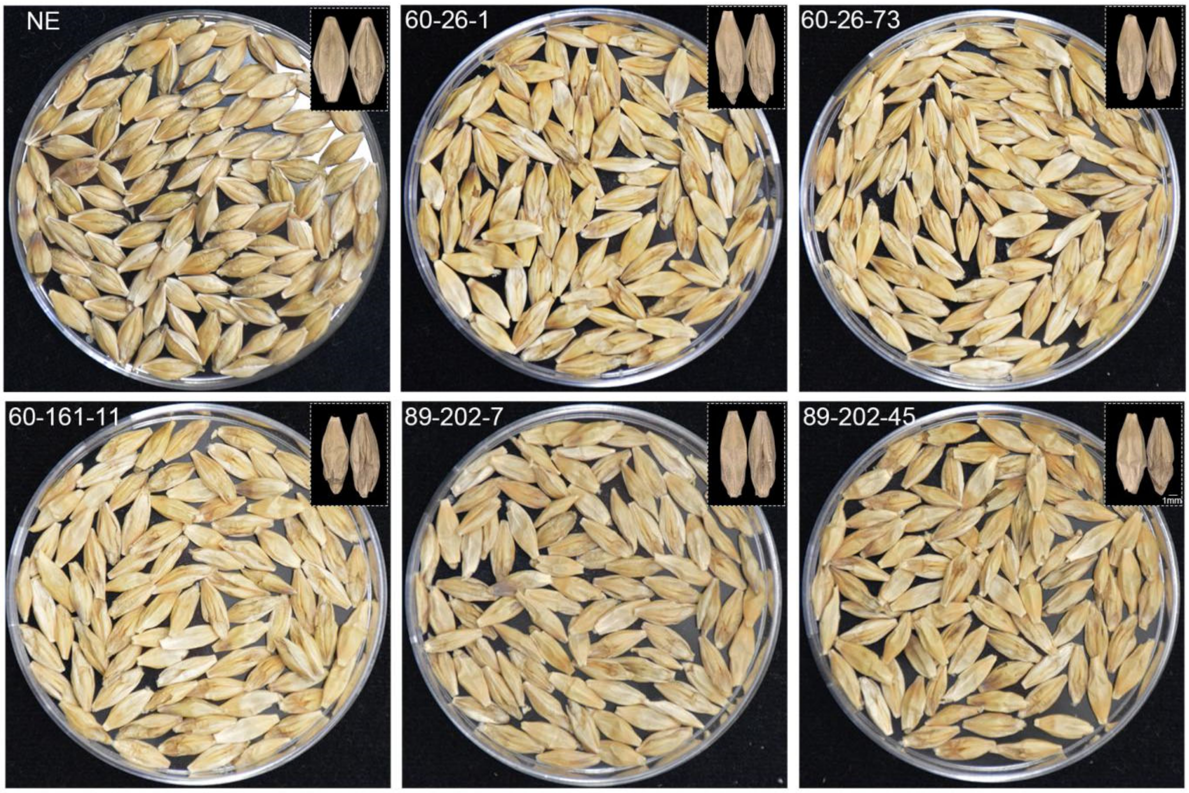


**Figure S1.** Stereoscopic and camera photographs of *ssIIa*/*ssIIIa*/*sbeIIa*/*sbeIIb* $T_3$ mutant grains. The line numbers of the mutant grains are indicated in the upper left corners of the corresponding images. NE, non-genome-edited line.

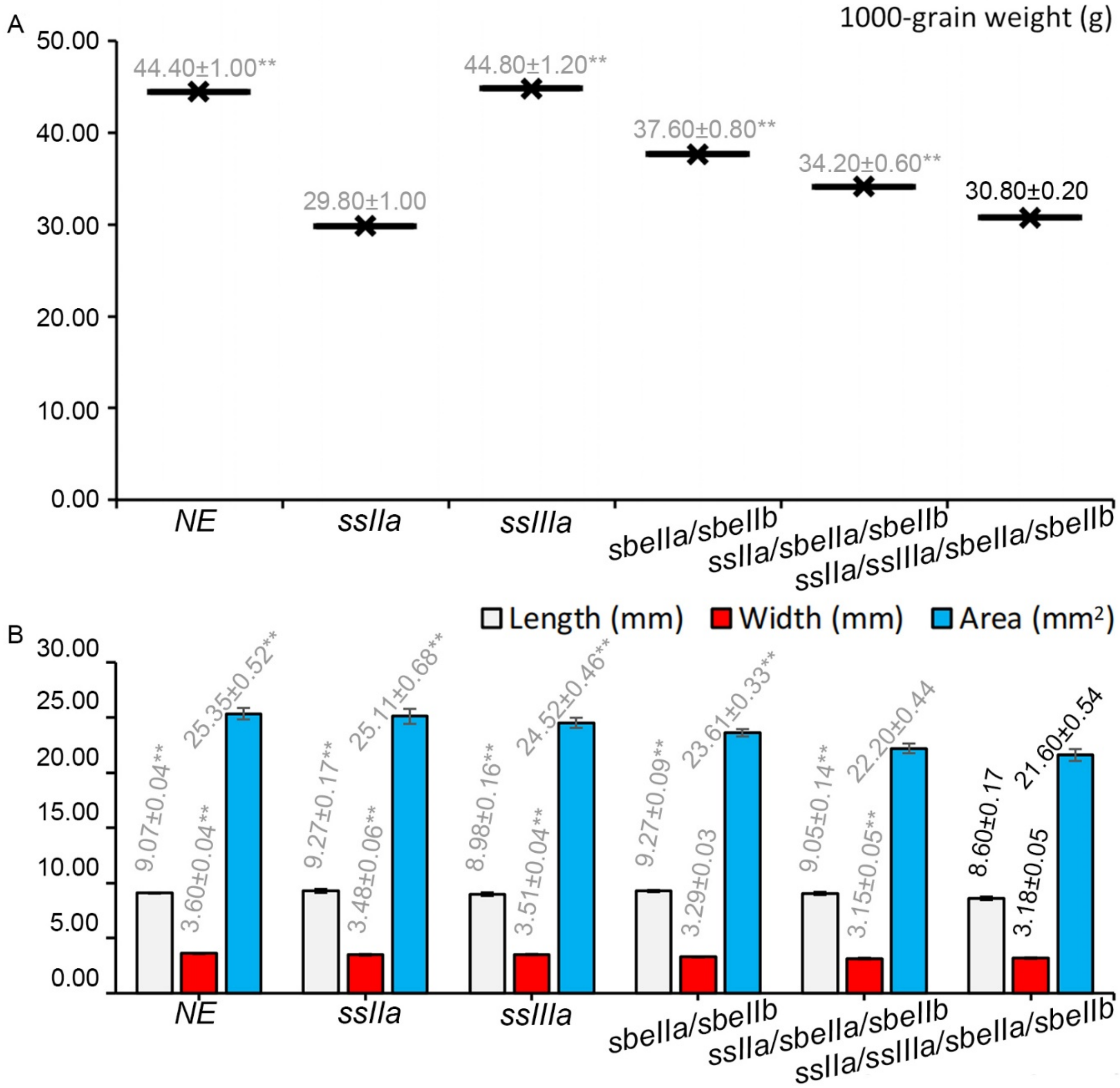


**Figure S2.** Grain weight and dimensions of $T_3$ mutant barley grains. (A) 1000-grain weight. (B) Grain length, width, and area. NE, non-genome-edited lines. The numbers above the columns represent the means of five biological replicates, along with their standard errors. The grey fonts indicate that the data has been published in a previous study [18]. Asterisks indicate the statistical significance between the *ssIIa/ssIIIa/sbeIIa/sbeIIb* mutant grains and other mutant grains, and NE determined by Student's t-tests (* at $P < 0.05$; ** at $P < 0.01$).

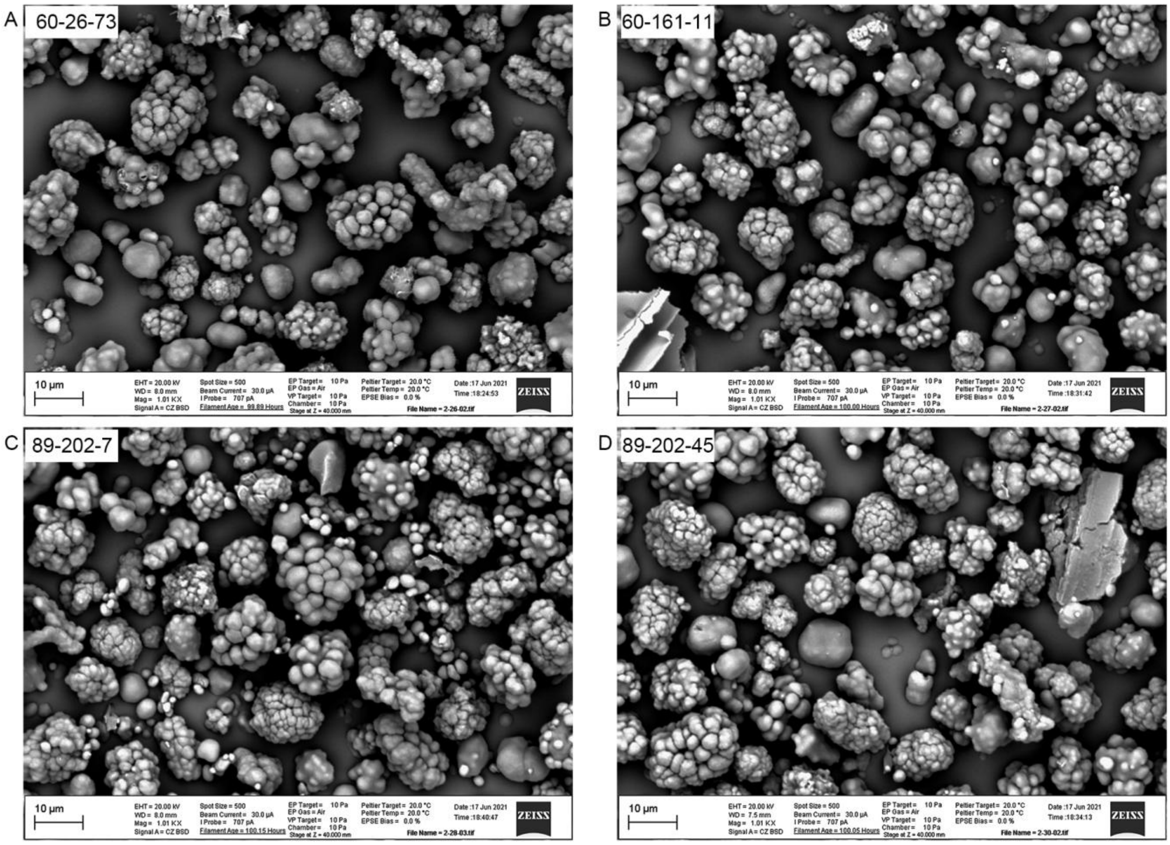


**Figure S3.** Starch granule morphologies in different *ssIIa/ssIIIa/sbeIIa/sbeIIb* mutant lines. Starch granule morphologies in mutant lines 60-26-73 (A), 60-161-11 (B), 89-202-7 (C) and 89-202-45 (D). Bars indicating 10 µm are shown on each micrograph.

**Table S1.** Differences in starch gelatinisation temperatures between *ssIIa/ssIIIa/sbeIIa/sbeIIb* and other barley mutants.

| Genotype | Gelatinisation Peak | | | | Amylose-Lipid Dissociation | | | |
|---|---|---|---|---|---|---|---|---|
| | Onset Temp (°C) | Peak Temp (°C) | End Temp (°C) | ΔH (J/g) | Onset Temp (°C) | Peak Temp (°C) | End Temp (°C) | ΔH (J/g) |
| *ssIIa/ssIIIa/sbeIIa/sbeIIb* | 61.18 ± 0.09 | 68.02 ± 0.39 | 74.88 ± 1.64 | 0.47 ± 0.26 | 91.78 ± 1.05 | 98.77 ± 1.34 | 105.68 ± 1.07 | 1.47 ± 0.18 |
| *NE* | *58.02 ± 0.73*** | *63.27 ± 0.58*** | *69.39 ± 1.03*** | *6.20 ± 0.31*** | *97.70 ± 2.51*** | *105.74 ±1.37*** | *114.10 ± 1.56*** | *0.74 ± 0.42* |
| *ssIIa* | *51.92 ± 0.33*** | *58.80 ± 0.97*** | *65.79 ± 1.65*** | *0.37 ± 0.09* | *94.00 ± 1.91* | *104.52 ± 1.26*** | *114.02 ± 0.86*** | *1.59 ± 0.37* |
| *ssIIIa* | *59.68 ± 0.53*** | *66.86 ± 0.61* | *74.76 ± 1.42* | *5.20 ± 0.27*** | *96.21 ± 1.67** | *104.55 ± 1.07*** | *113.36 ± 1.42*** | *0.90 ± 0.34* |
| *sbeIIa/sbeIIb* | *64.60 ± 0.71*** | *71.14 ± 2.50*** | *77.02 ± 3.67* | *0.37 ± 0.14* | *89.55 ± 1.96* | *98.45 ± 1.87* | *107.42 ± 0.93* | *1.68 ± 0.65* |
| *ssIIa/sbeIIa/sbeIIb* | *63.79 ± 0.66*** | *74.76 ± 0.90*** | *82.95 ± 0.42*** | *0.93 ± 0.31* | *95.20 ± 2.11* | *103.57 ± 1.89*** | *110.63 ± 1.45*** | *0.76 ± 0.26* |

The data underlined indicates that the data were published in a previous study [18]. Asterisks indicate a significant difference between the *ssIIa/ssIIIa/sbeIIa/sbeIIb* mutant and other mutants and NE determined by Student's t-tests (* at P < 0.05; ** at P < 0.01). NE, non-genome-edited lines.
